# hUCMSCs Mitigate LPS-Induced Trained Immunity in Ischemic Stroke

**DOI:** 10.3389/fimmu.2020.01746

**Published:** 2020-09-11

**Authors:** Yi-wei Feng, Cheng Wu, Feng-yin Liang, Tuo Lin, Wan-qi Li, Ying-hua Jing, Pei Dai, Hui-xian Yu, Yue Lan, Zhong Pei, Guang-qing Xu

**Affiliations:** ^1^Guangdong Key Laboratory for Diagnosis and Treatment of Major Neurological Diseases, National Key Clinical Department and Key Discipline of Neurology, Department of Neurology, The First Affiliated Hospital, Sun Yat-sen University, Guangzhou, China; ^2^Department of Neurology, Huashan Hospital, Fudan University, Shanghai, China; ^3^Department of Rehabilitation Medicine, School of Medicine, Guangzhou First People's Hospital, South China University of Technology, Guangzhou, China; ^4^Department of Rehabilitation Medicine, Guangzhou First People's Hospital, Guangzhou, China; ^5^Department of Rehabilitation Medicine, Beijing Tiantan Hospital, Capital Medical University, Beijing, China; ^6^China National Clinical Research Center for Neurological Diseases, Beijing, China

**Keywords:** innate immune memory, mesenchymal stem cell, stroke, microglia, H3K4me1

## Abstract

Innate immune memory is a part of the innate immune system that facilitates the elimination of pathogens. However, it may exacerbate neuropathology. In this study, we found that innate immune memory is detrimental in stroke, because it promotes the acute immune response and exacerbates ischemic infarcts. Mesenchymal stem cell therapy has been widely studied for its therapeutic potential in various diseases including stroke, but whether it diminishes innate immune memory has not been studied. Here, our study demonstrates that, after the activation of innate immune memory by lipopolysaccharide, mesenchymal stem cell therapy can diminish innate immune memory though down-regulation of H3 methylation and subsequently protect against stroke. Our results demonstrate that innate immune memory is detrimental in stroke, and we describe a novel potential therapeutic target involving the use of mesenchymal stem cells to treat stroke patients.

## Introduction

Stroke is the leading cause of death worldwide. The immune system in the brain has an important role in stroke pathophysiology. Immune dysfunction is detrimental, whereas immune modulation improves stroke outcomes (1, 2).

Immune memory has long been believed to exist only in the adaptive immune system. However, increasing evidence indicates that innate immune system also has memory (3, 4). Recently, innate immune memory (trained immunity) has been proposed to play a key role in neurological diseases. Initial inflammatory stimuli can induce epigenetic reprograming, such as histone modification, in which chromatin is made more accessible to the transcriptional machinery when subsequent immune insults occur. For example, peripheral injection of lipopolysaccharide (LPS) induces trained immunity in microglia, and trained microglia have been found to exacerbate ischemic brain damage 1 month after LPS challenge (5). Trained immunity is a long-lasting event, thus potentially explaining why patients with high risk factors for stroke, such as atherosclerosis and hypertension, are highly susceptible and vulnerable to stroke infarcts (6, 7). Therefore, the influence of trained immunity on stroke should be examined, and therapies should be identified to rectify this unwanted epigenetic rewiring.

Mesenchymal stem cells (MSCs) are multipotent, self-renewing cells, and MSC therapy has great potential in the treatment of neurological diseases (8, 9). The therapeutic aspects of MSCs for stroke patients have been intensively studied, and many MSC therapies have achieved great success (10–12). However, limited studies have focused on its therapeutic potential in modulating brain inflammation preceding stroke, which may be mediated by innate immune memory.

In this study, we established an LPS-induced mouse model of trained immunity in the central nervous system (CNS) and investigated the therapeutic potential of MSCs in diminishing trained immunity and thus ameliorating stroke outcomes.

## Materials and Methods

### Animals

C57BL/6J male mice (6–8 weeks, 20–25 g) were provided by the Sun Yat-sen University Medical Experimental Animal Center (Guangzhou, China). Animals were kept in a temperature- and humidity-controlled specific-pathogen-free laboratory with a 12/12-h light–dark cycle. Animal experimental procedures were performed in accordance with the Sun Yat-sen University Committee on the Care and Use of Animals.

### Experimental Design and Drug Administration

All the experimental groups were randomized, and all outcome analysis was carried out by independent investigators blinded to the treatment conditions and mouse types. Randomization was performed before the surgical procedure by the random number generator in GraphPad.

For LPS administration, mice were intraperitoneally (i.p.) injected with bacterial LPSs (from *Salmonella enterica* serotype *typhimurium*; Sigma) at the dose of 500 μg per kg of body weight.

For FK228 administration, mice were i.p. injected with FK228 (S3020; Shanghai Selleck Chemicals Co., Ltd.) at the dose of 20 μg per kg of body weight.

All experimental groups were randomized, and all outcome analysis was performed by independent investigators blinded to the treatment conditions and mouse types. Randomization was performed before the surgical procedure with the random number generator in GraphPad Prism 7.0 (GraphPad Software, Inc., San Diego, CA).

### Human Umbilical Cord MSC Ethical Approval and Isolation

Umbilical cords were sampled from healthy women who delivered babies by cesarean section at the Department of Obstetrics from the First Affiliated Hospital of Sun Yat-sen University. Each donor had been confirmed not to have infectious disease, pregnancy complications, hepatitis B virus, human immunodeficiency virus, or syphilis. Approval was granted by the donors, and all procedures were in accordance with the guideline of the Medical Ethics Committee of the Health Bureau.

The isolation of human umbilical cord MSCs (hUCMSCs) was performed as previously described (13). Briefly, the umbilical cord was placed in ice-cold phosphate-buffered saline (PBS), and the arteries and veins of the umbilical cord were discarded. Umbilical cords were carefully minced into small fragments, and the pieces were digested at 37°C for 3 h with 10 mL of 0.62 Wünsch units/mL collagenase I. The same volume of Dulbecco modified eagle medium (DMEM) as the digested fluid was added to stop the digestion, and the digested fluid was passed through a 70-μm strainer and then centrifuged to collect the cells. Human umbilical cord MSCs were cultured in DMEM/nutrient mixture F-12 with 10% fetal bovine serum and incubated at 37°C in a humidified, 5% CO_2_ atmosphere.

### Photothrombotic Stroke Model

After anesthesia with a combination of ketamine (0.12 mg/g, i.p.) and xylazine (0.01 mg/g, i.p.), the skull was exposed with a middle incision of the skin, and a 2.0-mm aperture in the posterior parietal cortex (4.0 mm posterior and 3.0 mm left of the bregma) was left for further illumination. Rose Bengal (10 mg/mL, dissolved in saline) was injected via the tail vein at a dose of 0.03 mg/g body weight. Then, the skull was illuminated for 10 min with a two-photon microscope with blue light (450–500 nm) focused on the aperture. After the illumination, skin was sutured, and the mice were kept on a warm heating pad and allowed to recover.

### Intravenous hUCMSC Administration

Human umbilical cord MSCs between passages three and five were collected, and ~10^6^ hUCMSCs/20 g were suspended in 0.2 mL saline and intravenously injected into mice at 0.1 mL/min.

### Microglia Isolation

The isolation of microglia was performed as previously described (14). Briefly, mice were anesthetized, and brains were collected and placed in ice-cold PBS as quickly as possible. Brains were carefully minced into small fragments, and the digestion of the brain was performed at 37°C for 10 min with papain (3 mg/mL) and DNase I. The digested tissue was centrifuged, and the cells were collected and resuspended in 37% Percoll. The resuspended cells were placed in 30% Percoll, and then 70% Percoll was added as the upper layer. The tubes were centrifuged at 3,000 rpm for 20 min without interruption. Cells were collected between the 30 and 37% Percoll and resuspended in Dulbecco phosphate-buffered saline PBS (~2 × 10^7^/mL). CD11b^+^ positive magnetic isolation was performed with a STEMCELL EasySep isolation kit (18,970, EasySep™ Mouse CD11b Positive Selection Kit II; STEMCELL, Canada). Isolated cells were lysed in radioimmunoprecipitation assay buffer (RIPA) and subjected to Western blot analysis.

### Western Blot Analysis

Mice were sacrificed and perfused with ice-cold saline. The ischemic cortex was sampled and homogenized by sonication in RIPA buffer. Equal masses of protein were subjected to Western blot analysis.

The following primary antibodies were used: anti–β-actin (1:1,000, 4,970; Cell Signaling Technology, USA), anti–IL-1β (1:1,000, 31,202; Cell Signaling Technology, USA), anti–IL-6 (1:1,000, 12,912; Cell Signaling Technology, USA), anti-H3K4me1 (1:1,000, 5,326; Cell Signaling Technology, USA); and anti-H3 (1:1,000, 4,499; Cell Signaling Technology, USA). The following secondary antibodies were used: goat–anti–rabbit horseradish peroxidase–linked antibody (1:1,000, 7,074; Cell Signaling Technology, USA).

### Immunofluorescence and Nissl Staining

Mice were euthanized and then perfused with ice-cold saline and fixed using 4% formaldehyde. After dehydration by 30% sucrose, coronal brain slices (20 μm thick) from infarcted cortices were sectioned using a frozen microtome (CM1950; Leica, Germany) at intervals of 200 μm to produce consecutive frozen sections.

For immunofluorescence, slices were first permeabilized by 0.1% Triton X-100 in PBS for 15 min and then blocked with PBS containing 5% bovine serum albumin for 1 h. Primary antibodies diluted in blocking buffer were added to the slices and incubated at 4°C overnight. Slices were then washed three times with PBS and incubated for 30 min at room temperature with the secondary antibody and then washed three more times with PBS. Slices were finally mounted with a DAPI mounting medium (Sigma-Aldrich) for subsequent observations.

The following antibodies were used in this experiment: anti-iba1 (1:200, ab5076; Abcam, USA), anti-CD16/32 (1:100, ab228971; Abcam, USA), anti-CD206 (1:100, ab64693; Abcam, USA), donkey–anti-rabbit IgG H&L (Alexa Fluor® 488) (1:500, ab150073; Abcam, USA), and donkey–anti-goat IgG H&L (Alexa Fluor® 555) (1:500, ab150130; Abcam, USA).

For Nissl staining, the sections were hydrated in 1% toluidine blue at 37°C overnight and washed with double distilled water. After being soaked in dimethylbenzene for 5 s, sections were sealed in Permount medium and covered by a coverslip. The infarct volume (mm^3^) was used to determine the infarct size in Image J (National Institutes of Health, USA).

### Statistical Analysis

Data were analyzed in GraphPad Prism 7.0 (GraphPad Software, Inc.). Data are represented as mean ± SD. Different treatment groups were evaluated with *t*-test or two-way analysis of variance (ANOVA) with Tukey test for multiple comparisons to determine differences among individual groups. A probability of *p* < 0.05 was considered indicative of significant differences between groups. Regardless of the method used, the results are equivalent in magnitude and statistically significant. Sample size was calculated a priori on the basis of a power of 0.95 and an α of 0.05. Detailed calculations are presented in the figure legends.

## Results

### Peripheral LPS Before Stroke Exacerbates Ischemic Brain Damage

**Figure 1:**
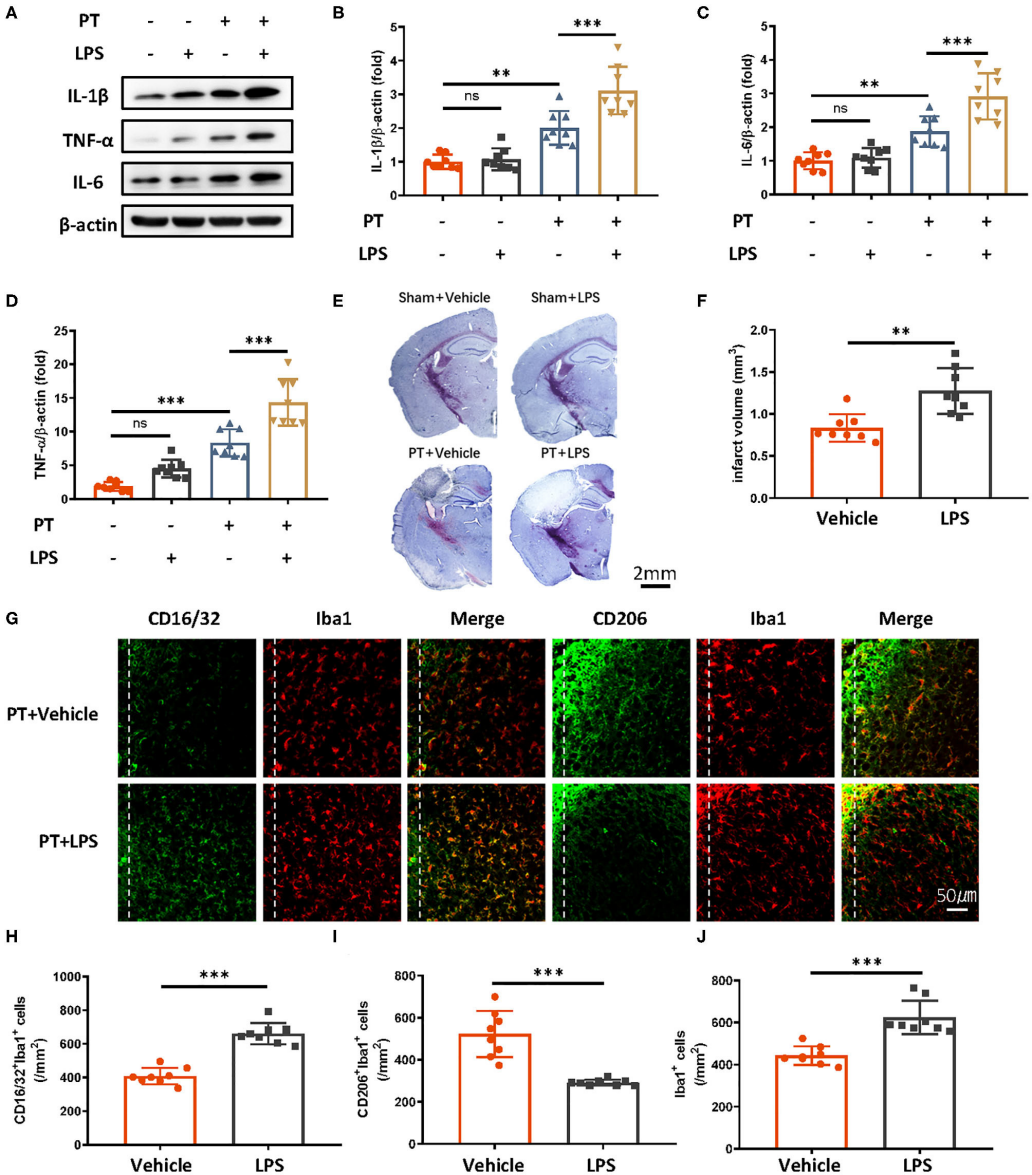
Peripheral LPS administration before stroke exaggerates ischemic brain damage. **(A–D)** Immunoblots **(A)** and quantitative analysis **(B–D)** showing that peripheral LPS administration before stroke promotes the production of proinflammatory cytokines. Lipopolysaccharide was i.p. administered; 4 weeks later, photothrombotic stroke (PT) was induced. Infarct samples were collected 24 h later. Interleukin 1β **(B)**, IL-6 **(C)**, and TNF-α **(D)** significantly increased after LPS stimulation (*n* = 8 mice per group). **(E,F)** Nissl staining **(E)** and quantitative analysis **(F)** showing that peripheral LPS treatment before stroke exacerbates infarct volume 7 days after stroke onset (*n* = 8 mice per group). **(G–J)** Representative immunofluorescence **(G)** and quantitative analysis **(H–J)** showing that LPS treatment preceding stroke elevated CD16/32-positive microglia, increased the number of microglia surrounding parainfarct region, and diminished CD206-positive microglia 7 days after photothrombotic stroke (*n* = 8 mice per group). Data were collected and analyzed with one-way ANOVA with Tukey multiple-comparisons test (ns, not significant; **p < 0.01; ***p < 0.001). Error bars, SD. Scale bars, 2 mm (“–” no corresponding treatment, “+” corresponding treatment, or administration).

Peripheral administration of low-dose LPS can stimulate immune responses in the brain. To test whether LPS-induced innate immune memory might exacerbate secondary stroke outcomes, we injected mice with low-dose of LPS (500 μg/kg, i.p.), and secondary photothrombotic stroke was induced 1 month later. At 24 h after stroke onset, the mice were sacrificed and perfused with ice-cold saline. The infarct cortex was sampled for Western blot analysis. One month after LPS treatment, the priming of LPS induced significantly higher IL-1β, IL-6, and TNF-α production 24 h after the subsequent stroke (Figures 1A–D), and infarct size was also exacerbated by the LPS priming (Figures 1E,F). Because microglia polarization (M1/2) indicates a distinct inflammatory phenotype of microglia, as M1 microglia are proinflammatory and M2 microglia are immunosuppressive, we observed microglia phenotype 7 days after photothrombotic stroke. We found that CD16/32-positive microglia (M1) and the number of microglia surrounding the parainfarct region were increased by LPS priming (Figures 1E–J). Meanwhile, CD206-positive microglia showed significant decrease (Figures 1G,I).

### Innate Immune Memory Mediates LPS-Induced Exacerbation of Stroke Outcomes

**Figure 2:**
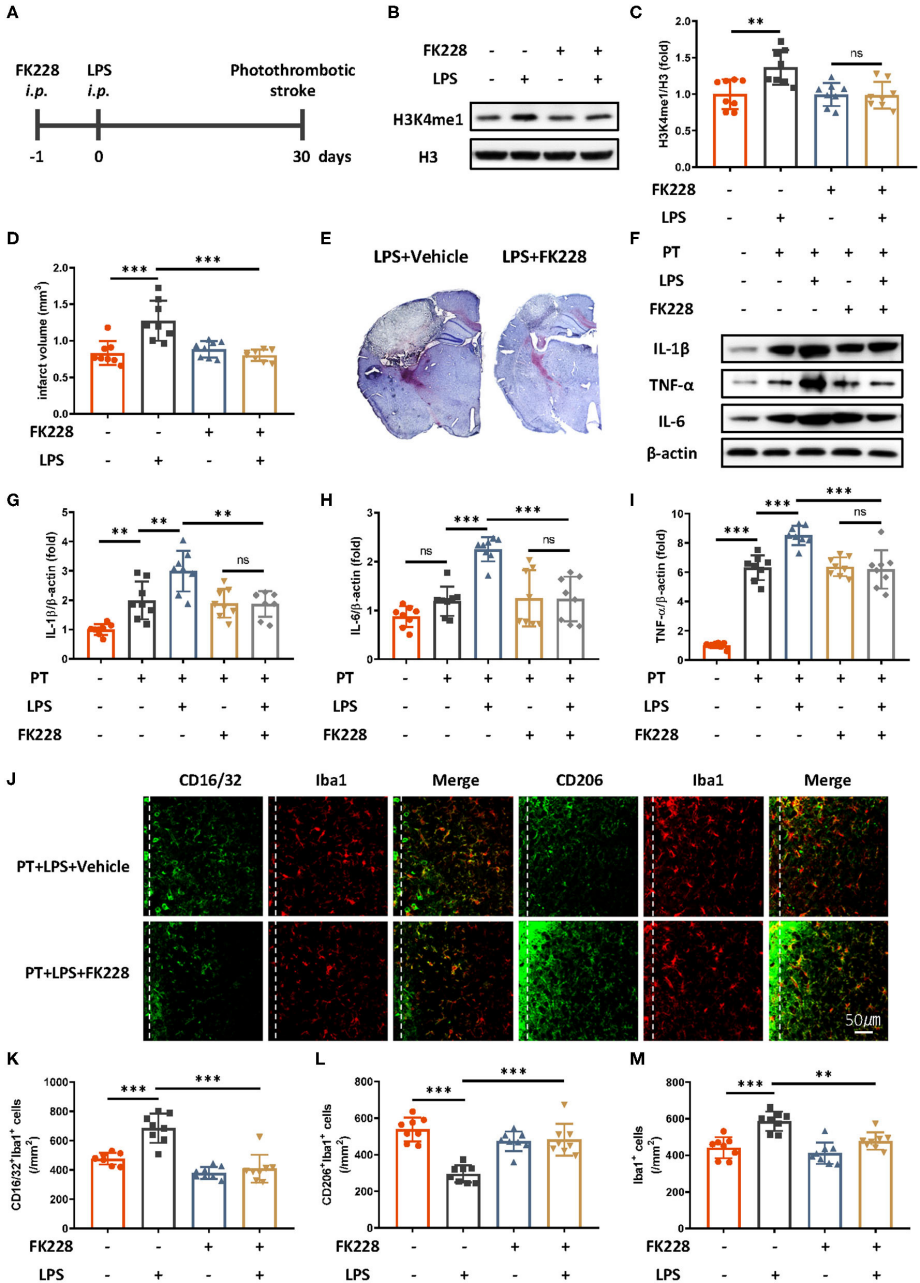
HDAC I/II inhibitor treatment attenuates ischemic brain damage via inhibition of LPS-induced innate immune memory. **(A)** Schematic diagram of experimental design. **(B,C)** Immunoblot **(B)** and quantitative analysis **(C)** showing H3K4me1 changes after LPS stimulation. Lipopolysaccharide induced significantly greater expression of H3K4me1, and this effect was abolished by treatment with FK228, an HDAC inhibitor (*n* = 8 mice per group). **(D)** Quantitative analysis of changes in infarct volume, showing that LPS-induced augmentation of infarcts was abolished by FK228 treatment (*n* = 8 mice per group). **(E)** Representative Nissl staining showing that LPS-induced elevation of infarct size was abolished by the FK228 treatment. **(F–I)** Immunoblots **(F)** and quantitative analysis **(G–I)** showing that LPS-induced increases in proinflammatory cytokines were abolished by FK228 treatment. Interleukin 1β **(G)**, IL-6 **(H)**, and TNF-α **(I)** were significantly increased by LPS stimulation and diminished by FK228 treatment (*n* = 8 mice per group). **(J–M)** Representative immunofluorescence **(J)** and quantitative analysis **(K–M)** showing that LPS treatment preceding stroke elevated CD16/32-positive microglia **(K)**, increased the number of microglia surrounding parainfarct region **(M)**, and diminished CD206-positive microglia **(L)** 7 days after photothrombotic stroke (*n* = 8 mice per group). Data were collected and analyzed with one-way ANOVA with Tukey multiple-comparisons test (ns, not significant; **p < 0.01; ***p < 0.001). Error bars, SD (“–” no corresponding treatment, “+” corresponding treatment, or administration).

Microglia are the main cell type in the brain producing proinflammatory cytokines during stroke. Previous research has proposed that the mechanism of innate immune memory in the brain is the epigenetic rewiring of H3K4me1 in the microglia (15). To investigate whether the LPS-induced exacerbation of the acute inflammatory response in ischemic mice occurs though the regulation of innate immune memory, we isolated microglia in the brain through CD11b^+^ positive magnetic isolation and examined changes in H3K4me1 after LPS administration. H3K4me1 increased dramatically in mice receiving LPS (Figures 2B,C). Further blocking of H3 methylation by FK228, an HDAC I/II inhibitor able to cross the blood–brain barrier (Figure 2A), abolished LPS-induced epigenetic reprogramming, alleviated the aggravated inflammatory response, and decreased the infarct size (Figures 2D–I). Moreover, LPS-induced elevation of CD16/32-positive microglia was diminished by the FK228, which was accompanied by the increased CD206-positive microglia and alleviated number of microglia surrounding parainfarct region after FK228 treatment (Figures 2J–M). Together, these results demonstrated that low-dose LPS induced trained immunity in microglia, thereby further exacerbating ischemic outcomes.

### MSCs Diminish LPS-Induced Innate Immune Memory and Are Protective in Ischemic Stroke

**Figure 3:**
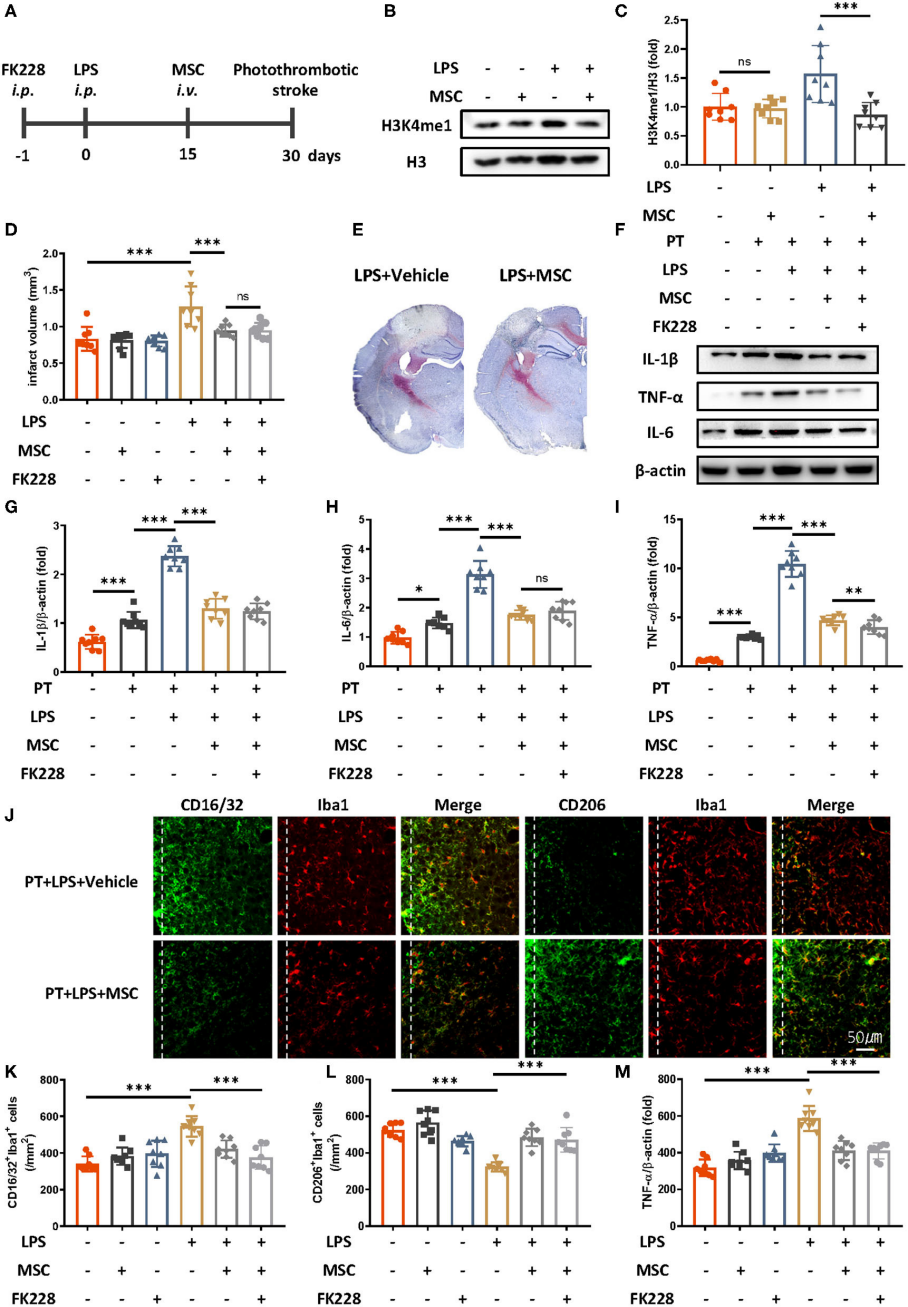
Mesenchymal stem cells diminish LPS-induced innate immune memory and are protective in ischemic stroke. **(A)** Schematic diagram of experimental design. **(B,C)** Immunoblot **(B)** and quantitative analysis **(C)** showing that MSCs mitigate LPS-induced H3K4me1 elevation. Lipopolysaccharide induced significantly greater expression of H3K4me1, and this effect was mitigated by the administration of MSCs (*n* = 8 mice per group). **(D)** Quantitative analysis of infarct volume changes, showing that the LPS-induced augmentation of infarcts was diminished by MSCs (*n* = 8 mice per group). **(E)** Representative Nissl staining showing that LPS-induced elevation of infarct size was rectified by the MSC treatment. **(F–I)** Immunoblots **(F)** and quantitative analysis **(G–I)** showing that the LPS-induced increase in proinflammatory cytokines was rectified by MSCs. Interleukin 1β **(G)**, IL-6 **(H)**, and TNF-α **(I)** were significantly increased by LPS stimulation and diminished by MSCs. FK228 treatment further proved the specificity of MSCs in reacting this abnormal immune memory (*n* = 8 mice per group). **(J–M)** Representative immunofluorescence (J) and quantitative analysis **(K–M)** showing that the LPS-induced elevation of CD16/32-positive microglia **(K)** aggravated number of microglia surrounding parainfarct region **(M)**, and down-regulation of CD206-positive microglia **(L)** was rectified by MSC treatment 7 days after photothrombotic stroke (*n* = 8 mice per group). Data were collected and analyzed with one-way ANOVA with Tukey multiple-comparisons test (ns, not significant; *p < 0.05; **p < 0.01; ***p < 0.001). Error bars, SD (“–” no corresponding treatment, “+” corresponding treatment, or administration).

Because MSC therapy has shown promise in promoting brain repair in various brain diseases (11, 16, 17), we wondered whether MSC treatment might relieve LPS-induced innate immune memory. Mesenchymal stem cells were intravenously administered 2 weeks after LPS injection and 2 weeks before ischemic stroke (Figure 3A). Previous research has indicated that the existing MSCs are <10% 2 weeks after injection, and the influence of MSCs themselves is minimal (18). We then examined the epigenetic changes after MSCs administration (Figures 3B,C). Compared with LPS treatment only, LPS and MSC treatment robustly alleviated microglial H3K4me1 modification in mice. In addition, the previously exacerbated acute inflammatory response and infarct outcomes due to LPS were diminished by MSC treatment (Figures 3D–I). The elevated expression of CD16/32-positive microglia, increased number of microglia surrounding parainfarct region, and down-regulated expression of CD206-positive microglia were also rectified by the administration of MSCs (Figures 3J–M).

No significant changes in inflammatory cytokines, expression of CD16/32 of CD 206 positive microglia, number of microglia surrounding parainfarct region, and infarct size were observed between MSCs-treated mice and MSCs-FK228–injected mice, thus indicating that the specificity of MSC treatment was due to alleviated epigenetic reprogramming (Figures 3D–M).

## Discussion

In this study, we examined the role of MSCs in LPS-induced trained immunity in ischemic stroke. We found that LPS activates innate immune memory in the brain, and this trained effect, although epigenetic rewiring of H3K4me1, exacerbates ischemic infarcts by efficiently initiating an acute inflammatory response. However, administration of MSCs diminishes LPS-induced trained immunity and rescues ischemic infarcts. Given that intravenous administration of MSCs has been found to be safe in both clinical and experimental studies, the therapeutic effects of MSCs on trained immunity provide additional evidence supporting the application of MSCs in ischemic stroke treatment.

Currently, the limited therapeutic potential of stroke treatment has caused studies to increasingly emphasize stroke prevention as the most effective strategy to decrease health consequences (19, 20). In this study, we first investigated the risk effect of trained immunity in the development of stroke pathology and observed the therapeutic effect of MSCs in modifying this abnormal trained effect.

### Trained Immunity in CNS Shows Detrimental Effect on Neurological Pathology

The recently discovered immune training indicates that the innate immune system may recognize some proinflammatory stimuli and respond more efficiently in the presence of secondary stimuli. The purpose of innate immune memory is to eliminate pathogens more efficiently, but in the CNS, immune training may exacerbate neuropathology in some aspects (5). For example, a single administration of LPS in APP/PS1 mice robustly increases β-amyloid deposition 6 months later, thus indicating that the innate immune memory may last at least 6 months (5). The detrimental aspects of trained immunity may be due to the long-lived properties of microglia: the consistent activation of the immune system may induce a vicious circle promoting neuropathology. In this study, we investigated the influence of trained immunity on subsequent stroke. Lipopolysaccharide stimulation before stroke onset promoted the production of proinflammatory cytokines and resulted in exacerbated infarct volume, thus indicating that prior inflammatory stimuli long before a stroke can have detrimental effects. Our follow-up study further indicated that the deterioration associated with prior LPS exposure was due to the epigenetic histone modification of H3K4me1 in microglia, which is a hallmark of trained immunity. Our study therefore highlights that the epigenetically activated immune system in the CNS may exacerbate stroke outcomes.

Notably, trained immunity can be activated not only by microbial products, such as LPS, but also by some stroke risk factors (21). For example, a high fat diet has been reported to induce epigenetic reprogramming in the innate immune system in an NLRP3-depedent manner (21). In addition, small vascular disease characterized by hypoperfusion in the brain is a high risk factor for cognitive impairment after stroke. Previous studies have demonstrated that chronic hypoxia epigenetically changes the microglia, and subsequent inflammatory stimuli can further promote the detrimental influence of microglia on neurons (22). Moreover, studies have demonstrated that hypertension can prime the inflammatory environment and result in poorer stroke outcomes. Therefore, patients at high risk should be notified, and effective therapies should be developed to rectify these abnormal epigenetic changes.

### Therapeutic Potential of MSCs in Rectifying Trained Immunity in CNS

Mesenchymal stem cells are multipotent, self-renewing cells, and the potential of MSC therapy has been investigated in the treatment of cardiovascular and neurodegenerative disorders (23, 24). The therapeutic aspects of MSCs in curing stroke have been identified (17). Mesenchymal stem cells exhibit their therapeutic potential mainly through modulation of the inflammatory environment, stimulating endogenous neurogenesis and angiogenesis and illuminating glial scars via the production of paracrine factors (11). Post-stroke MSC therapy has been extensively studied, but the therapeutic window of efficiency for MSCs is relatively narrow and limited. Mesenchymal stem cells' anti-inflammatory, neuroprotective, and angiogenic functions are effective only in relatively early or acute stages of stroke; however, most patients diagnosed with stroke are often in late chronic stages (25). Given the previously recognized role of innate immune memory and the anti-inflammatory effect of MSCs, we investigated the therapeutic potential of MSCs in mice with immune training. Consistently with our hypothesis, MSC treatment alleviated H3K4 methylation in microglia, the inflammatory response was mitigated, and the infarct size decreased.

Until now, limited researches have focused on the role of MSCs in epigenetically reprograming target cells. Our study figured out one putative mechanism for MSCs to epigenetically regulate trained microglia by inhibiting H3K4 methylation. And these results support the therapeutic potential of MSC therapy in diminishing epigenetic reprograming and minimizing the subsequent inflammatory response in ischemic infarcts.

It is true that MSC therapy for stroke patient was mainly restricted to the post-stroke treatment. Because most stroke episodes are unpredictable, suggesting that stem cell therapy may be more appropriate as a regenerative biologic. But post-stroke treatment has limited effect as infarct region have been already formed, and the most efficient way to cure stroke patient is thrombolytic therapy in the acute phase of stroke. Moreover, with the advent of diagnostic tools designed to identify at-risk stroke patients based on family history, genetics, and comorbidity factors (e.g., diabetes, hypertension), we have more intervention period, and contemplating stem cells as preventive/protective therapeutics is therefore appearing more realistic. In our study, we used LPS to initiate trained immunity in microglia. But it should be noticed that innate immune memory could also be activated by hypertension, diabetes, and high-fat diet as we mentioned before. So, MSCs could not only be used among post-stroke patient but also could be developed as a prophylactic therapy to rectify the trained immunity activated by these risk factors.

Although the extensive mechanism through which MSCs diminish epigenetic reprograming remains to be elucidated, and biomarkers to probe immune training in microglia have yet to be developed, our research provides novel insight into the therapeutic potential of MSCs in curing stroke patients. We hope that our research will provide a basis for focusing on the prevention of stroke rather than post-stroke treatment.

## Data Availability Statement

The raw data supporting the conclusions of this article will be made available by the authors, without undue reservation.

## Ethics Statement

The animal study was reviewed and approved by Sun Yat-sen University Committee on the Care and Use of Animals.

## Author Contributions

YF drafted the manuscript. YF, CW, WL, TL, and FL accomplished the experiment. GX, ZP, YF, and YL designed the experiment. YJ, PD, and HY provided technical support. YL, ZP, and GX provided financial support. All authors contributed to the article and approved the submitted version.

## Conflict of Interest

The authors declare that the research was conducted in the absence of any commercial or financial relationships that could be construed as a potential conflict of interest.

## Abbreviations

ANOVA: analysis of variance
CNS: central nervous system
DNase I: deoxyribonuclease I
hUCMSCs: human umbilical cord mesenchymal stem cells
H3K4me1: histone H3, mono methylated at lysine 4
H3K4me3: histone H3, trimethylated at lysine 4
HDAC I/II: histone deacetylases I/II
LPS: lipopolysaccharide
MSCs: mesenchymal stem cells
NLRP3: NOD-, LRR-, and pyrin domain-containing protein 3
PBS: phosphate-buffered saline
PT: photothrombotic stroke
RIPA: radioimmunoprecipitation assay buffer
SD: standard deviation
USA: United States of America.
